# Associations of sleep quality, nutrition, community participation, cognitive function, depressive symptoms, and fall risk in older adults with dynapenic abdominal obesity: a cross-sectional study

**DOI:** 10.1186/s12877-026-07022-8

**Published:** 2026-01-26

**Authors:** Chin-Chih Lin, Yu-Chen Su, Hsiao-Chi Tsai, Yen-Hung Chen, Shu-Fang Chang

**Affiliations:** 1 Public Health Bureau of Hsinchu County Government, Hsinchu, Taiwan; 2 Nursing Department, Hsin Sheng Junior College of Medical Care and Management, Taoyuan, Taiwan; 3 Department of Nursing, Cardinal Tien Hospital, Xindian District, New Taipei City, Taiwan; 4 Demartment of Information Management, National Taipei University of Nursing and Health Sciences, Taipei, Taiwan; 5 School of Nursing, College of Nursing, National Taipei University of Nursing and Health Sciences, 365 Ming Te Road, Pei-Tou, Taipei, 112 Taiwan

**Keywords:** Dynapenic abdominal obesity, Sleep quality, Nutritional status, Community participation, Cognitive function, Depression, Falls risk

## Abstract

**Background:**

Dynapenic abdominal obesity (DAO) is known to increase the risk of physical decline and falls in older adults. However, it is still unclear how different factors—such as sleep quality, nutrition, social participation, cognition, and mood—work together to influence this risk. The aim of this study was to identify factors associated with fall risk—specifically sleep quality, nutritional status, community participation, cognitive function, and depressive symptoms—among community-dwelling older adults with dynapenic abdominal obesity.

**Methods:**

We conducted a cross-sectional study with 180 adults aged 65 and above. Validated questionnaires were used to measure sleep quality, nutritional status, community participation, cognitive function, depressive symptoms, and fall risk. Data were analyzed using t-tests, ANOVA, and logistic regression.

**Results:**

Although poor sleep showed differences in the univariate analysis, it did not remain significant in the multivariable model. Older adults with malnutrition, lower community participation, cognitive decline, or depressive symptoms were more likely to have a higher fall risk. Logistic regression further indicated that nutritional status (OR = 7.03, *p* = .013), community participation (OR = 0.91, *p* = .031), cognition (OR = 6.18, *p* = .028), and depressive symptoms (OR = 9.23, *p* = .008) were significant predictors.

**Conclusion:**

Fall risk in older adults with DAO is influenced by a mix of physical, psychological, and social factors. Community nurses and other health professionals should routinely check nutrition, cognitive function, and emotional health to identify those at higher risk early and provide more holistic, preventive care that supports safe aging.

## Background

The rapid shift toward an aging global population has brought increasing attention to the health and functional needs of older adults. United Nations projections indicate that by 2050, adults aged 65 and older will comprise about 16% of the world’s population—an increase from roughly 10% in 2022 [1]. Taiwan is undergoing a similar demographic transition and is expected to enter a super-aged phase by 2025, with nearly one in five citizens aged 65 years or above [2].

With advancing age, many individuals experience progressive losses in muscle strength, balance, and mobility, which increase vulnerability to falls. In recent years, attention has turned to dynapenic abdominal obesity (DAO), a condition characterized by reduced muscle strength alongside central adiposity [3]. DAO is typically defined by low handgrip strength combined with abdominal obesity based on waist circumference thresholds, and is estimated to affect 10%–12% of community-dwelling older adults [4, 5].

Falls remain a major public health concern and are identified by the World Health Organization as a leading cause of injury and mortality, accounting for more than 680,000 deaths annually [6]. Older adults with DAO appear to face particularly elevated risks. For example, Gadelha et al. [7] reported that approximately 40% of Brazilian older adults with DAO had experienced a fall, while data from the English Longitudinal Study of Ageing show higher risks of recurrent falls and fall-related injuries among individuals with DAO compared with those without the condition [8].

Despite growing evidence on fall risk in older adults, limited research has specifically examined dynapenic abdominal obesity (DAO) and its association with fall risk in Asian populations. Differences in body composition patterns, muscle mass distribution, and waist circumference cutoffs across ethnic groups suggest that findings derived from Western cohorts may not be fully applicable to Asian contexts. In addition, few studies have investigated how multiple health-related factors—including sleep quality, nutritional status, community participation, cognitive function, and depressive symptoms—collectively relate to fall risk among older adults with DAO.

Therefore, this study aimed to examine the associations between these multidimensional health-related factors and fall risk in community-dwelling older adults with dynapenic abdominal obesity in Taiwan. Fall risk was operationalized using the Falls Risk for Older People in the Community (FROP-Com) scale as a validated outcome measure, and logistic regression analyses were conducted to explore associations between fall risk status and these health-related factors, rather than to replicate or replace the FROP-Com assessment itself.

## Methods

### Study design and population

This cross-sectional study involved 180 community-dwelling older adults with dynapenic abdominal obesity, recruited from community centers and local health stations in northern Taiwan between January and June 2024. The planned sample size was determined using G*Power 3.1.9.7, applying the parameters reported by Kobayashi [9] (power = 0.80, effect size = 0.15, α = 0.05). Participants were eligible if they were 65 years or older and met the diagnostic criteria for DAO. After obtaining informed consent, data were gathered through structured, face-to-face interviews along with standardized physical assessments conducted in accordance with established community health procedures.

### Definition of dynapenic abdominal obesity (DAO)

Dynapenic abdominal obesity (DAO) was defined as the co-occurrence of dynapenia and abdominal obesity. To identify dynapenia, we adopted the criteria proposed by Kobayashi et al. [9], which were developed and validated specifically for Asian community-dwelling older adults. Because Taiwanese older adults share similar anthropometric and body composition characteristics with other Asian populations, these criteria were considered more appropriate and culturally relevant for this study. In contrast, EWGSOP2 and AWGS 2019 are primarily designed for diagnosing sarcopenia, which requires low muscle mass; applying those standards may misclassify individuals who meet the conceptual definition of dynapenia—reduced muscle strength but preserved muscle mass. Therefore, Kobayashi’s criteria provide a more accurate framework for identifying dynapenia and abdominal obesity within an Asian context and are most suitable for the purposes of this study.

Under Kobayashi et al. [9] framework, dynapenia is characterized by reduced muscle strength—specifically, handgrip strength < 26 kg in men and < 18 kg in women—and/or slowed gait speed (< 0.8 m/s), while muscle mass remains within the normal range. Accordingly, muscle mass was assessed using bioelectrical impedance analysis (BIA), and normality was determined based on appendicular skeletal muscle mass index values of ≥ 7.0 kg/m² for men and ≥ 5.7 kg/m² for women. Both handgrip strength and gait speed were assessed. Handgrip strength was measured using a calibrated dynamometer, and gait speed was assessed over a standardized walking distance according to established protocols.

Abdominal obesity was classified according to the waist circumference criteria commonly used in Taiwan, with values ≥ 90 cm for men and ≥ 80 cm for women considered indicative of central adiposity. These cutoffs align with recommendations from Taiwan Ministry of Health and Welfare and the Asia-Pacific Working Group, which better reflect the body composition and visceral fat distribution characteristics of Asian populations. These criteria were applied at the recruitment stage; therefore, all analyses were conducted among community-dwelling older adults with dynapenic abdominal obesity.

### Demographic characteristics

Demographic data included age, sex, educational level, marital status, body mass index (BMI), smoking, alcohol consumption, hearing, vision, chronic conditions, polypharmacy, fall history, and comorbidities.

### Muscle mass

Muscle mass was assessed using a bioelectrical impedance analysis (BIA) device (ACCUNIQ BC380, Korea), which estimates skeletal muscle and fat mass across five body regions (both arms, both legs, and the trunk). Previous studies have shown that BIA measurements strongly correlate with dual-energy X-ray absorptiometry (DEXA) results (*r* = .946, *p* < .05) in healthy adults [9]. The method is considered reliable, quick, noninvasive, and free of radiation exposure [10].

### Muscle strength

Handgrip strength (HGS) was measured using a TKK dynamometer (Japan) with a range of 5–100 kg. Previous study has demonstrated high test-retest reliability (0.98) and validity compared with the Jamar dynamometer (*r* = .94, *p* < .05) [9].

### Sleep quality

Sleep quality was evaluated using the Pittsburgh Sleep Quality Index (PSQI) [11], which captures multiple aspects of sleep behavior, including sleep duration, disturbances, latency, and efficiency. Participants scoring more than 5 on the PSQI were considered to have poor overall sleep quality. The Chinese version (CPSQI) has been validated among middle-aged and older adults, showing satisfactory internal consistency (Cronbach’s α = 0.82–0.83) and test–retest reliability (*r* = .85, *p* < .05) [12].

### Nutritional status

Nutritional status was assessed using the Mini Nutritional Assessment (MNA), an 18-item tool with total scores ranging from 0 to 30. A score of 24 or higher reflects adequate nutrition, 17–23.5 suggests risk of malnutrition, and below 17 indicates poor nutrition. The MNA is widely applied in geriatric populations and has demonstrated high validity and diagnostic accuracy [13].

### Community participation

Participants in community life were measured using a 16-item Community Participation Scale developed by [14]. The instrument examines four dimensions: activity attendance, involvement, perceived control, and overall engagement. Each item is rated on a 4-point Likert scale, where higher scores indicate greater engagement in community activities. The internal reliability of this measure was acceptable (Cronbach’s α = 0.67).

### Cognitive function

Cognitive function was assessed using the Mini-Mental State Examination (MMSE), a 30-point screening tool evaluating orientation, attention, recall, and language abilities. Higher scores indicate better cognitive performance [15]. The Chinese version of the MMSE (C-MMSE), translated and validated by Guo et al. (1988), has demonstrated good reliability among older adults (Cronbach’s α = 0.89) [16] and is widely used in non-commercial academic research in Taiwan and other Chinese-speaking regions. In this study, only total MMSE scores were recorded, and no copyrighted or proprietary MMSE materials were reproduced.

### Depression

Depressive symptoms were measured using the 15-item Geriatric Depression Scale (GDS-15), which asks respondents to provide yes/no answers regarding emotional status and activity levels. Scores range from 0 to 15, with higher scores indicating more depressive symptoms [17]. The GDS-15 has been shown to have good psychometric reliability among older adults (Cronbach’s α = 0.86) [18].

### Fall risk

Fall risk was determined using the Taiwan version of the FROP-COM scale [19]. This 21-item instrument evaluates individual and environmental factors contributing to fall risk. The scale demonstrates strong content validity (CVI = 0.94) and moderate-to-high inter-rater reliability (*r* = .65–1.00, *p* < .05). Fall risk was classified according to the original FROP-Com scoring system and the Taiwan validation study, with total scores categorized as low risk (0–14), moderate risk (15–22), or high risk (23–45) [19]. For the purpose of binary logistic regression analyses, the moderate and high risk categories were combined, and fall risk was dichotomized into low risk (0–14) and moderate-to-high risk (≥ 15).

### Statistical analysis

Statistical analyses were performed using SPSS version 25.0 (IBM Corp., Armonk, NY, USA). Descriptive statistics, including frequencies, means, and standard deviations, were used to summarize participant characteristics. Univariate group comparisons between fall risk categories were performed using independent t-tests or chi-square tests, as appropriate. These analyses assessed differences by fall-risk level in sociodemographic characteristics, sleep quality, nutritional status, community participation, cognitive function, and depressive symptoms.

Binary logistic regression was conducted to examine factors associated with moderate-to-high fall risk. Variables showing significant associations with fall risk in the univariate analyses were entered into the multivariable model. Although some of the examined factors may conceptually overlap with domains partially captured by the FROP-Com scale, they were measured using independent, standardized instruments and analyzed as distinct constructs. The regression analyses were therefore intended to provide complementary information on multidimensional health-related correlates of fall risk beyond the composite FROP-Com score, rather than to replicate or replace the FROP-Com assessment. Multicollinearity among independent variables was assessed using variance inflation factors (VIFs), with all values below 5, indicating no evidence of problematic multicollinearity. Statistical significance was set at *p* < .05.

## Results

### Demographics, sleep quality, nutritional status, community participation, cognitive function, depressive symptoms, and fall risk

Among the 180 community-dwelling older adults with dynapenic abdominal obesity (DAO), most participants were women (58.3%) with a mean age of 73.24 ± 5.46 years (range = 65–93). The majority had completed high school (33.9%), were married (71.1%), and lived with family members (87.2%). Nearly all participants had children (95.0%) and did not receive social assistance (91.7%). Buddhism was the most common religion (51.1%). Most took one to three types of medication (62.2%), and the majority did not smoke (78.3%) or drink alcohol (86.1%). A large proportion (74.4%) reported at least one chronic condition, with hypertension being the most frequent (76.1%) (Table 1).

**Table 1:** Participants’ characteristics in sociodemographics, sleep quality, nutritional status, community participation, cognitive function, and depressive symptoms (*N* = 180)

Characteristics	n		%		Mean	SD
Gender						
Male	75		41.7			
Female	105		58.3			
Age					73.24	5.46
65–69 years	54		30.0			
70–74 years	49		27.2			
75 years and above	77		42.8			
Education level						
Illiterate	6		3.3			
Elementary school	50		27.8			
Junior high school	42		23.3			
Senior high school	61		33.9			
College or above	21		11.7			
Occupation						
Retired	14		7.8			
Industry	9		5.0			
Commerce	11		6.1			
Agriculture	14		7.8			
Military/public service	5		2.8			
Self-employed	17		9.4			
Other	110		61.1			
Marital status						
Never married	2		1.1			
Married	128		71.1			
Cohabiting	5		2.8			
Divorced	10		5.6			
Widowed	35		19.4			
Number of children					2.81	1.31
No children	9		5.0			
Has children	171		95.0			
Living arrangement						
Lives with family	157		87.2			
Lives alone	22		12.2			
Other	1		0.6			
Social assistance						
No social assistance	165		91.7			
Receives social assistance	15		8.3			
Religious affiliation						
No religion	32		17.8			
Buddhism	92		51.1			
Taoism	43		23.9			
Christianity	5		2.8			
Catholicism	2		1.1			
Folk religion	4		2.2			
Other religions	2		1.1			
Number of medications						
No medications	34		18.9			
1–3 medications	112		62.2			
4–6 medications	27		15.0			
≥ 7 medications	7		3.9			
Smoking status						
Non-smoker	141		78.3			
Smoker	39		21.7			
Alcohol use						
No alcohol use	155		86.1			
Drinks alcohol	25		13.9			
Chronic disease					1.16	0.93
No chronic disease	46		25.6			
With chronic disease	134		74.4			
Types of chronic disease (multiple responses)						
Hypertension	102		76.1			
Diabetes	44		32.8			
Hyperlipidemia	39		29.1			
Stroke	9		6.7			
Cancer	6		4.5			
Other diseases	8		6.0			
Body mass index (BMI)					25.35	2.87
Normal weight	59		32.8			
Overweight	74		41.1			
Obese	47		26.1			
Sleep quality					8.05	4.012
Good sleep quality	51		28.3		3.22	1.4
Poor sleep quality	129		71.7		9.96	2.96
Nutritional assessment					26.05	2.81
Normal nutrition (≥ 24)	152		84.4		26.98	1.66
Malnourished or at risk (< 24)	28		15.6		21.04	2.45
Community participation					37.36	10.14
Attendance	36		20		2.53	0.75
Involvement	38		21		2.43	0.72
Control	52		29		1.62	0.70
Full engagement	54		30		2.59	0.69
Cognitive function					25.77	3.99
Normal cognition (24–30)	138		76.6		27.57	1.88
Cognitive impairment (< 24)	42		23.3		19.83	3.27
Depressive symptoms					2.57	2.44
No depressive symptoms (≤ 5)	159		88.3		1.9	1.49
Depressive symptoms (> 6)	21		11.7		7.67	2.2
Fall risk					7.62	5.53
Low risk (0–14)	155		86.1		5.86	3.33
Moderate-to-high risk (15–45)	25		13.9		18.48	3.69

The mean body mass index (BMI) was 25.35 kg/m², with the largest group classified as overweight (41.1%). In terms of sleep quality, most participants (71.7%) reported poor sleep. Nutritional status was generally adequate, with 84.4% classified as well-nourished (MNA ≥ 24). For community participation, the highest mean score was observed for full engagement (M = 2.59, SD = 0.69), whereas the lowest was for sense of control (M = 1.62, SD = 0.70). Regarding cognitive function, 76.7% scored within the normal range (24–30), and 23.3% showed signs of impairment. Only 11.7% had depressive symptoms (GDS > 5). As for fall risk, the majority were classified as low risk (86.1%), while 13.9% were at moderate to high risk (Table 1).

### Differences in the demographic characteristics, sleep quality, nutritional status, community participation, cognitive function, depressive symptoms, and fall risk among participants

Significant associations were observed between fall risk and several demographic variables, including age group (χ² = 14.51, *p* < .001), education level (χ² = 11.88, *p* = .003), number of medications (χ² = 9.76, *p* = .003), and presence of chronic disease (χ² = 4.70, *p* = .030).

Sleep quality also differed notably by fall risk group. Poor sleepers were more frequent among those at moderate-to-high risk (18.6%) than among good sleepers (2.0%) (χ² = 8.47, *p* = .004), with significantly lower overall PSQI scores (t = -4.29, *p* < .001) (Table 2). Nutritional status followed a similar pattern—over half of the malnourished or at-risk group (53.6%) were at moderate-to-high risk compared with only 6.6% of the well-nourished participants (χ² = 11.47, *p* = .030), and their MNA scores were significantly lower (t = 5.73, *p* < .001). Community participation was also poorer among those with greater fall risk. Scores for attendance (t = 4.33, *p* < .001), involvement (t = 5.34, *p* < .001), control (t = 5.21, *p* < .001), full engagement (t = 3.48, *p* < .001), and overall participation (t = 6.12, *p* < .001) were all lower in the moderate-to-high risk group (Table 2).

**Table 2:** Differences by fall-risk level in sociodemographics, sleep quality, nutritional status, community participation, cognitive function, and depressive symptoms

Characteristics	Fall riskLow risk(*n* = 155)	Fall risk Moderate-to-high risk(*n* = 25)	t / χ²	*p*
Gender			0.48	0.489
Male	63 (41.0)	12 (48.0)		
Female	92 (59.0)	13 (52.0)		
Age			14.51	0.001
65–69 years	54 (34.8)	2 (8.0)		
70–74 years	42 (27.0)	7 (28.0)		
75 years and above	59 (38.2)	16 (64.0)		
Education level			11.88	0.003
Elementary school or below	41 (26.5)	15 (60.0)		
Junior high school	40 (25.8)	2 (8.0)		
College or above	74 (47.7)	8 (32.0)		
Occupation			1.67	0.196
Retired	104 (67.1)	20 (80.0)		
Not retired	51 (32.9)	5 (20.0)		
Marital status			1.47	0.225
Without a partner	38 (24.5)	9 (36.0)		
With a partner	117 (75.5)	16 (64.0)		
Children			1.23	0.615
No children	9 (5.8)	0 (0.0)		
Has children	146 (94.2)	25 (100.0)		
Living arrangement			1.35	0.328
Lives with family	137 (88.4)	20 (80.0)		
Alone or others	18 (11.6)	5 (20.0)		
Social assistance			1.40	0.232
No social assistance	144 (92.9)	21 (84.0)		
Receives social assistance	11 (7.1)	4 (16.0)		
Religious affiliation			1.32	0.328
No religion	137 (88.4)	20 (80.0)		
With religion	18 (11.6)	5 (20.0)		
Number of medications			9.76	< 0.01
No medications	33 (21.3)	1 (4.0)		
1–3 medications	101 (65.2)	11 (44.0)		
≥ 4 medications	21 (13.5)	13 (52.0)		
Smoking status			3.51	0.061
Non-smoker	125 (80.6)	16 (64.0)		
Smoker	30 (19.4)	9 (36.0)		
Alcohol use			3.59	0.054
No alcohol use	137 (88.4)	18 (72.0)		
Drinks alcohol	18 (11.6)	7 (28.0)		
Chronic disease			4.70	0.030
No chronic disease	44 (28.4)	2 (8.0)		
With chronic disease	111 (71.6)	23 (92.0)		
BMI classification			4.83	0.090
Normal weight	53 (34.2)	6 (24.0)		
Overweight	66 (42.6)	8 (32.0)		
Obese	36 (23.2)	11 (44.0)		
BMI (Body Mass Index)	25.28 ± 0.90	25.77 ± 2.69	0.78	0.435
Sleep quality category			8.47	0.004
Good sleep quality	50 (32.3)	1 (4.0)		
Poor sleep quality	105 (67.7)	24 (96.0)		
Total score of sleep quality	7.63 ± 3.99	10.64 ± 3.12	4.29	< 0.01
Nutritional status category			9.47	< 0.01
Normal nutrition (≥ 24)	142 (91.6)	10 (40.0)		
Malnourished or at risk (< 24)	13 (8.4)	15 (60.0)		
Total score of nutritional status	26.66 ± 2.08	22.28 ± 0.73	5.73	< 0.01
Community participation				
Attendance	10.49 ± 2.92	7.80 ± 2.63	4.33	< 0.01
Involvement	15.05 ± 0.34	11.72 ± 2.59	5.34	< 0.01
Control	5.08 ± 2.14	3.52 ± 1.23	5.21	< 0.01
Full engagementa	7.99 ± 2.09	6.48 ± 1.50	3.48	< 0.01
Total score of community participation	38.62 ± 0.09	29.52 ± 6.24	6.12	< 0.01
Cognitive function status			45.02	< 0.01
Normal cognition (24–30)	132 (85.2)	6 (24.0)		
Cognitive impairment (< 24)	23 (14.8)	19 (76.0)		
Total score of cognitive function	26.57 ± 3.28	20.80 ± 4.45	6.21	< 0.01
Depressive symptoms			18.47	< 0.01
No depressive symptoms (≤ 5)	147 (94.8)	12 (48.0)		
Depressive symptoms (> 6)	8 (5.2)	13 (52.0)		
Total score of depressive symptoms	2.09 ± 1.94	5.56 ± 3.06	5.50	< 0.01

Footnote: Values are presented as n (%). Percentages are calculated by column. Group comparisons were performed using chi-square tests

Cognitive impairment was markedly more prevalent among those at moderate-to-high fall risk (45.2%) than among participants with normal cognition (4.3%) (χ² = 45.02, *p* < .001), and their MMSE scores were significantly lower (20.80 ± 4.45 vs. 26.57 ± 3.28, t = 6.21, *p* < .001). Likewise, depressive symptoms were more common in the higher-risk group (61.9%) compared with those without symptoms (7.5%) (χ² = 18.47, *p* < .001), with higher mean GDS-15 scores (5.56 ± 3.06 vs. 2.09 ± 1.94, t = -5.50, *p* < .001) (Table 2).

### Predictive factors for fall risk in older adults with dynapenic abdominal obesity

Binary logistic regression was performed to identify factors associated with moderate-to-high fall risk, using variables that showed significant associations in the univariate analyses. Among demographic characteristics, age, education level, number of medications, and chronic disease differed significantly between fall-risk groups in the univariate analyses and were therefore entered into the multivariable logistic regression model. However, after adjustment for other covariates, none of these demographic variables remained statistically significant in the final model.

In the regression analyses, odds ratios were estimated using the low fall risk group as the reference category for the outcome variable. For categorical predictors, reference groups were defined as elementary school or below for education level, no medications for number of medications, absence of chronic disease, good sleep quality, good nutritional status, normal cognitive function, and absence of depressive symptoms. In the final multivariable model, four variables remained significantly associated with fall risk: nutritional status, overall community participation, cognitive function, and depressive symptoms. Poorer nutritional status, as indicated by lower Mini Nutritional Assessment scores, was associated with higher odds of moderate-to-high fall risk (OR = 7.03, *p* = .013). Greater community participation was associated with lower odds of fall risk (OR = 0.91, *p* = .031), suggesting a protective association of social engagement. Participants with cognitive impairment had higher odds of moderate-to-high fall risk compared with those with normal cognitive function (OR = 6.18, *p* = .028). In addition, depressive symptoms were significantly associated with increased odds of moderate-to-high fall risk (OR = 9.23, *p* = .008) (Table 3).

**Table 3:** Predictors of fall risk among community-dwelling older adults with DAO (binary logistic regression)

Predictors	B	SE	OR (95% CI)	*p*
Age	0.05	0.08	1.05 (0.91–1.22)	0.493
Education level				
Junior high vs. Elementary or below	-2.05	1.36	0.13 (0.01–1.85)	0.131
Senior high or above vs. Elementary or below	-0.17	0.77	0.84 (0.19–3.77)	0.823
Number of medications				
1–3 medications vs. No medications	0.13	1.72	1.13 (0.04–33.04)	0.942
≥ 4 medications vs. No medications	1.58	1.79	4.84 (0.15–60.46)	0.377
Chronic disease (yes vs. no)	-0.12	1.42	0.89 (0.06–14.36)	0.935
Sleep quality (poor vs. good)	1.59	1.30	4.90 (0.38–63.14)	0.223
Nutritional status (at risk/poor vs. good)	1.95	0.79	7.03 (1.50–33.02)	0.013*
Overall community participation	-0.10	0.05	0.91 (0.83–0.99)	0.031*
Cognitive function (impaired vs. normal)	1.82	0.83	6.18 (1.22–31.17)	0.028*
Depressive status (yes vs. no)	2.22	0.83	9.23 (1.80–47.39)	0.008**
Constant	-5.86	5.54		

Note: *B* Regression coefficient, *SE* Standard error, *OR * Odds ratio

* *p* < .05, ** *p* < .01

## Discussion

Our findings showed that in this Taiwanese older participants with DAO were mostly female, with a mean age of 73.24 years. Many had completed high school, were overweight, and frequently reported hypertension. Notably, our results echoed those described by de Oliveira Maximo et al. [20], who found that women and individuals with hypertension were more likely to exhibit DAO in a Brazilian cohort. The slightly lower BMI observed in the present study may reflect regional variations in dietary patterns and lifestyle habits. Most participants reported taking one to three medications and did not smoke or consume alcohol, a trend also seen in Araujo et al. [3].

Poor sleep quality was reported by 71.7% of participants, closely matching rates found among outpatients with dynapenia [5] and among institutionalized older adults [21]. However, the percentage was higher than that reported by Di Simone et al. [22] among hospitalized older adults in Lithuania. This difference may be related to variations in population health profiles, care routines, and environmental factors between community and hospital settings. Nutritional status in this sample was largely within normal range, supporting the observations of Atmis [23]. In contrast, Lv et al. [24] found that hospitalized older adults with DAO were more prone to malnutrition, likely due to disease-related appetite changes and reduced nutrient absorption.

More than half of the participants regularly took part in community activities. This finding corresponds to the community engagement levels reported by Luo et al. [25] and Lin [26]. Tsai and Chang [5], however, noted lower participation among dynapenic outpatients, which may relate to greater physical limitations in that group. Cognitive impairment was observed in 23.3% of participants—lower than the rates reported by Lv et al. [24] and Oba et al. [27], but higher than those of Hatanaka et al. [28]. Differences in health conditions, nutritional patterns, and daily activity could help explain these variations. Depressive symptoms were found in 11.7% of participants, similar to Carvalho et al. [29], yet lower than Qian et al. [30], whose participants were younger and likely faced additional social and occupational pressures. A considerable proportion of participants were classified as having moderate-to-high fall risk (13.9%), based on the Taiwan FROP-Com scale, an outcome comparable to Zhang et al. [8] but somewhat higher than Dowling et al. [31]. Differences in recall period and age structure could account for this variation.

In the present study, fall risk was significantly associated with several demographic and health-related characteristics, including age, education level, medication use, and chronic disease burden. Participants classified as having moderate-to-high fall risk tended to be older, less educated, and to report a greater number of comorbidities and medications, findings that are consistent with previous reports by Smith et al. [6] and Lv et al. [24]. In addition, the number of children was associated with fall risk, suggesting that family interaction and social support may contribute to maintaining safety and daily functioning in later life, as also noted by Moncayo-Hernandez et al. [32].

Sleep quality showed a significant association with fall risk in the univariate analyses, a finding that aligns with prior studies indicating that fragmented or insufficient sleep is linked to an increased risk of falls among older adults [33, 34]. However, this association did not remain significant after adjustment for other covariates in the multivariable model. This attenuation may be explained by the close interrelationships among sleep quality, depressive symptoms, and cognitive function. Sleep disturbance frequently co-occurs with depressive symptoms and cognitive impairment, both of which are well-established risk factors for falls among older adults [24]. After accounting for these psychological and cognitive factors, the independent association between sleep quality and fall risk was reduced. These findings suggest that the effect of sleep quality on fall risk may be indirect and partially mediated through psychological and cognitive pathways. Nevertheless, due to the cross-sectional design of this study, causal relationships and mediation mechanisms cannot be definitively established and warrant further investigation in longitudinal studies.

In the current study, nutritional status was an important factor. Those with poorer nutritional scores or at risk of malnutrition showed a higher incidence of falls, echoing the reports of Lv et al. [24] and Eckert et al. [35], who emphasized the protective role of adequate nutrition in maintaining balance and muscle function. In addition, participants with limited community participation—particularly those with lower involvement and reduced sense of control—were more prone to falls, a relationship also highlighted by Wang et al. [36] and Moncayo-Hernandez et al. [32].

Our research found that cognitive decline and depressive symptoms both increased the likelihood of falls. Cognitive impairment was associated with gait instability and reduced coordination, as seen in Lv et al. [24] and Kuan et al. [37]. Likewise, depressive symptoms heightened fall risk, consistent with findings from Chao et al. [38] and Maximo et al. [39], who observed that depression can slow psychomotor responses, reduce motivation, and intensify fear of falling. In the multivariate model, nutritional status, community participation, cognitive function, and depressive symptoms remained significantly associated with fall risk. These findings suggest that fall risk in older adults with DAO arises from a multidimensional interaction among physiological, psychological, and social factors. Strengthening nutritional health, promoting social engagement, maintaining cognitive vitality, and managing depressive symptoms may collectively reduce fall risk in this vulnerable population. Early screening of these indicators within community health programs could support individualized prevention strategies and enhance healthy aging outcomes.

### Limitations

This study used a cross-sectional quantitative design; therefore, the findings reflect only the conditions of older adults with DAO at the time of data collection and do not permit causal interpretation or evaluation of temporal changes. Participants were recruited from a single community in northern Taiwan, which may limit the generalizability of the results to other regions or populations. In addition, the multivariable model included only variables that were significant in the univariate analyses, meaning that residual confounding cannot be fully excluded. Future studies incorporating larger and more geographically diverse samples, along with longitudinal or experimental designs, are recommended to strengthen external validity and provide a more comprehensive understanding of DAO-related fall risk in older adults.

## Conclusion

This study showed that nutritional status, community participation, cognitive function, and depressive symptoms were all closely related to fall risk among community-dwelling older adults with dynapenic abdominal obesity. Malnutrition, cognitive decline, depressive symptoms, and limited community engagement indicators related to fall risk. These results underscore that fall risk in this population is shaped by the combined effects of physical, psychological, and social factors rather than any single domain. Routine assessment of these indicators can help clinicians and community health professionals identify high-risk individuals early and guide the development of more holistic fall prevention strategies for older adults with DAO.

## Data Availability

The datasets generated and analyzed during the current study are not publicly available due to participant privacy restrictions but are available from the corresponding author upon reasonable request.
